# A weighted sparse coding model on product Grassmann manifold for video-based human gesture recognition

**DOI:** 10.7717/peerj-cs.923

**Published:** 2022-03-16

**Authors:** Yuping Wang, Junfei Zhang

**Affiliations:** 1 School of Statistics, Capital University of Economics and Business, Beijing, China; 2 School of Statistics and Mathematics, Central University of Finance and Economics, Beijing, China

**Keywords:** Product Grassmann manifold, Sparse coding, Video classification, Human gesture recognition

## Abstract

It is a challenging problem to classify multi-dimensional data with complex intrinsic geometry inherent, such as human gesture recognition based on videos. In particular, manifold structure is a good way to characterize intrinsic geometry of multi-dimensional data. The recently proposed sparse coding on Grassmann manifold shows high discriminative power in many visual classification tasks. It represents videos on Grassmann manifold using Singular Value Decomposition (SVD) of the data matrix by vectorizing each image in videos, while vectorization destroys the spatial structure of videos. To keep the spatial structure of videos, they can be represented as the form of data tensor. In this paper, we firstly represent human gesture videos on product Grassmann manifold (PGM) by Higher Order Singular Value Decomposition (HOSVD) of data tensor. Each factor manifold characterizes features of human gesture video from different perspectives and can be understood as appearance, horizontal motion and vertical motion of human gesture video respectively. We then propose a weighted sparse coding model on PGM, where weights can be understood as modeling the importance of factor manifolds. Furthermore, we propose an optimization algorithm for learning coding coefficients by embedding each factor Grassmann manifold into symmetric matrices space. Finally, we give a classification algorithm, and experimental results on three public datasets show that our method is competitive to some relevant excellent methods.

## Introduction

Human action/gesture recognition (Pareek & Thakkar, 2021) is a hot research area due to its wide applications such as human–computer interaction, robot control, security and survillance, sign language assistance, education, medical, etc. Roughly speaking, human actions /gestures convey intentional information by physical movement of body parts. Usually, the term “action” is considered with a higher complexity level comparing to the term “gesture” (Zhu et al., 2016). Researches for human gesture recognition are mainly divided into two categories: wearable device based techniques (Jung et al., 2015) and vision-based techniques (Ji et al., 2012). However, wearing devices requires users to carry special designed wearable sensors and sensors are usually quite expensive. For vision-based approaches, videos carry more information for gesture recognition than still images. Moreover, the number of available videos on the Internet significantly increased with the development of acquisition and storage device. Hence, video-based human gesture recognition (Ji et al., 2012; Chakraborty et al., 2018; Patil & Subbaraman, 2019) attracts more and more attentions.

For video-based human gesture recognition, each video is assigned to a class label and videos of the same class maybe acted by different person in different environment. It becomes more difficult for gesture recognition due to large variations, such as illumination, appearance, pose and scale. There exist variations even though for the same person. Therefore it is a challenging problem for video-based human gesture recognition. Basically, the key problems of video-based human gesture recognition are learning discriminative feature representations for a gesture video and designing an effective recognition method.

For feature representation, some researches focused on handcrafted approaches, such as HOG-3D (Klaser, Marszałek & Schmid, 2008), space–time interest point (Laptev, 2005), pose-based techniques (Carreira et al., 2016), motion-based techniques (Paul, Haque & Chakraborty, 2013), shape-based techniques (Vishwakarma & Kapoor, 2015). Some researches focused on learning-based approaches which can be roughly divided into non-neural network and neural network learning approaches. The latter approaches received good recognition performances because it is designed to mimic human nervous system biologically, such as 3D ConvNets (Baccouche et al., 2011; Tran et al., 2015; Feichtenhofer, Pinz & Wildes, 2016) and variational autoencoder(VAE) (Spurr et al., 2018; Chen et al., 2019). Millions of parameters need to be learned by training networks and large amounts of data are often required. For non-neural network learning approaches, subspace is a robust representation and had received good performance for many problems in computer vision field (Le et al., 2011; Sheng et al., 2019). The reason is that most data often have intrinsic subspace structure and can be regarded as samples of subspace. Moreover, subspace-based feature representation method can learn features directly from image or video data without hand-designed local feature. For investigating and representing the underlying intrinsic subspace structure, many subspace methods were proposed, such as linear subspace learning (PCA (Wold, Esbensen & Geladi, 1987), FLDA (Belhumeur, Hespanha & Kriegman, 1997; Mohammadzade, Sayyafan & Ghojogh, 2018)) and non-linear manifold learning (Isomap (Pless, 2003), LLE (Ge, Yang & Lee, 2008), LE (Luo, 2011)). As an excellent representative, Grassmann manifold received widely applications such as activity classification (Turaga & Chellappa, 2009), action recognition (Rahimi, Aghagolzadeh & Ezoji, 2019), face recognition (Huang et al., 2015) and so on.

For recognition methods, sparsity representation classification (SRC) had been shown to deliver notable results for various visual-based tasks, such as face recognition (Wright et al., 2008; Wright et al., 2010), subspace clustering (Elhamifar & Vidal, 2013). Furthermore, some weighted forms for sparse coding were proposed for various applications, such as image denoising (Xu, Zhang & Zhang, 2018), visual tracking (Yan & Tong, 2011) and saliency detection (Li, Sun & Yu, 2015). Although the SRC method and its extended models had good performance in many applications, they assumed data come from linear space. However, many multi-dimensional data may reside in a non-linear manifold space. So it is desire to explore the latent non-linear manifold structure of data. Recently, for Grassmann manifold representation of videos/image sets, many researches had been proposed for kinds of applications and received good performance. For instance, Harandi et al. (2015) proposed a sparse coding algorithm on Grassmann manifold for classification tasks such as gesture classification, scene analysis and dynamic texture classification; Wang et al. (2020) proposed a self-expression learning framework on Grassmann manifolds for video/image-set subspace clustering; Verma & Choudhary (2020) did Grassmann manifold discriminant analysis for hand gesture recognition from depth data; Souza et al. (2020a) proposed an enhanced Grassmann discriminant analysis framework for classifying motion sequences.

Although the Grassmann manifold can well reflect the non-linear structure of data, the single space representation methods lose some important information by vectorizing each image in videos. Naturally, video and image set can be represented in the form of data tensor. Tensor computing had been successfully applied to many visual-based application (Kim & Cipolla, 2008). Lui (2012) factorized a data tensor using Higher Order Singular Value Decomposition (HOSVD) and imposed each factorized element on a Grassmann manifold, then a video can be represented as a point on product Grassmann manifold (PGM). This representation yielded a very discriminating structure for action recognition. Wang et al. (2016) proposed a low rank representation model on PGM, which received good performance for clustering of videos or image sets. Wang et al. (2018) proposed an extrinsic least square regression on PGM for video-based recognition.

In this paper, we represent a human gesture video as a point on PGM. In brief, there are three factor Grassmann manifolds which can reflect appearance, horizontal motion and vertical motion of human gesture video respectively. In addition, the importance of these three aspects should be considered. Hence, we explore a weighted sparse coding method on PGM for video-based human gesture recognition. It is solved by minimizing the reconstruction error with a *l*_1_ −norm regularizer.

Our main contributions lie in the following three aspects:

(1) Extending SRC model on Grassmann manifold into product Grassmann manifold to deal with multi-dimensional data such as videos and image-sets.
(2) Discussing the different importance of three factor manifolds and proposing a weighted sparse coding model.
(3) Comparing with several classification methods on three datasets to show the effectiveness of our proposed method.

The rest of this paper is organized as follows: ‘Product Grassmann Manifold Representation for Data’ introduces product Grassmann manifold representation for data; ‘Weighted Sparse Coding on Product Grassmann Manifold’ gives a weighted sparse coding model on PGM; ‘Experiments’ shows experiments on different datasets, and experiment results show that the proposed method achieves considerable accuracy; ‘Computational Complexity’ analyzes the computational complexity of our proposed method; ‘Main Findings and Future Directions’ gives main findings and future directions.

## Product Grassmann manifold Representation for data

In the following paper, we use the mathematical symbols in Table 1 which are commonly used.

### Product Grassmann manifold

A point on Grassmann manifold $\mathcal{G}(p,d)$ is a *p*-dimensional subspace of ℝ^*d*^ (Absil, Mahony & Sepulchre, 2009). That means it can be spanned by any orthonormal basis **X** = [**x**_1_|**x**_2_|⋯|**x**_*p*_] ∈ ℝ^*d*×*p*^ and it is denoted as span(**X**). For the sake of convenience, we use the same symbol **X** to represent span(**X**). The distance of two points **X** and **Y** on Grassmann manifold can be defined as

\begin{eqnarray*}{d}_{g}(\mathbf{X},\mathbf{Y })=\parallel \Pi (\mathbf{X})-\Pi (\mathbf{Y }){\parallel }_{F}=\parallel \mathbf{X}{\mathbf{X}}^{T}-\mathbf{Y }{\mathbf{Y }}^{T}{\parallel }_{F} \end{eqnarray*}

where embedding mapping $\mathrm{&Pi;}:\mathcal{G}(p,d)&rarr; \mathrm{Sym}(d)$ is defined as Π(**X**) = **XX**^*T*^, and Sym(*d*) is the symmetric matrices space with order *d* (refer to Harandi et al., 2015). Product Grassmann manifold (PGM) $\mathcal{PG}$(*p*_1_, …, *p*_*M*_| *d*_1_, …, *d*_*M*_) is defined as

\begin{eqnarray*}\mathcal{PG}({p}_{1},\ldots ,{p}_{M}{|}{d}_{1},\ldots ,{d}_{M})=\mathcal{G}({p}_{1},{d}_{1})\times \cdots \times \mathcal{G}({p}_{M},{d}_{M}) \end{eqnarray*}

where the symbol × denotes Cartesian product, $\mathcal{G}({p}_{i},{d}_{i})$ (*i* = 1, …, *M*) is called factor manifold and *p*_*i*_(*i* = 1, ⋯, *M*) is called dimension of each factor manifold. A point on PGM is denoted as [**X**] = (**X**^1^, …, **X**^*M*^). The distance between two points [**X**] = (**X**^1^, …, **X**^*M*^) and [**Y**] = (**Y**^1^, …, **Y**^*M*^) on PGM is defined as weighted average distance of each factor Grassmann manifold

\begin{eqnarray*}{d}_{\mathcal{PG}}([\mathbf{X}],[\mathbf{Y }])=\sqrt{\sum _{m=1}^{M}{\omega }_{m}{d}_{g}^{2}({\mathbf{X}}^{m},{\mathbf{Y }}^{m})} \end{eqnarray*}

where each weight *ω*_*m*_(≥0) represents the importance of factor manifold $\mathcal{G}({p}_{m},{d}_{m})$ and $\sum _{m=1}^{M}{\omega }_{m}=1$.

### Data representation on PGM

In the real world, there exists many data with multi-dimensional structure. For example, video can be represented as tensor 𝒜 ∈ ℝ^*J*_1_×*J*_2_×*J*_3_^, where *J*_1_, *J*_2_ and *J*_3_ represent height, width and length of video respectively; Image set can be represented as tensor 𝒜 ∈ ℝ^*J*_1_×*J*_2_×*J*_3_^, where *J*_1_, *J*_2_ and *J*_3_ represent height, width and number of image set respectively; Light field can be represented as tensor 𝒜 ∈ ℝ^*J*_1_×*J*_2_×*J*_3_×*J*_4_^ (Wang & Zhang, 2020), where *J*_1_ and *J*_2_ represent angular resolution of light field, *J*_3_ and *J*_4_ represent spatial resolution of light field.

**Table 1:** Mathematical symbols in this paper.

Symbol	Description
**X**, **Y**, …	a matrix
**x**, **y**, …	a vector
$\mathcal{X},\mathcal{Y },&hellip; $	a tensor
*N*, *M*, *d*, *p*, …	scalar
**x**_*i*_, …	the *i*th column of matrix **X**
*x*_*ij*_, …	the (*i*, *j*)-th element of matrix **X**
**X** ^*T*^	the transpose of matrix **X**
Tr(⋅)	sum of the diagonal elements of a matrix
∥⋅∥_*F*_	$\parallel \mathbf{X}{\parallel }_{F}=\sqrt{\mathrm{Tr}({\mathbf{X}}^{T}\mathbf{X})}$
∥⋅∥_1_	∥**X**∥_1_ = ∑_*i*,*j*_|*x*_*ij*_|

**Figure 1:**
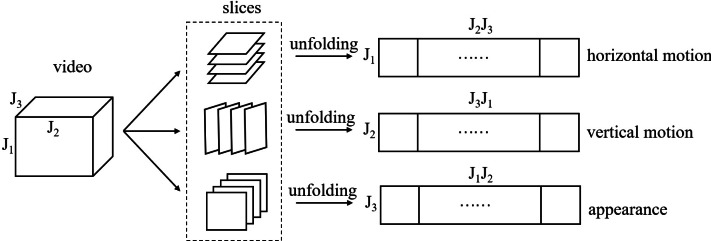
A schematic of matrix unfolding for a video tensor. *J*_1_, *J*_2_ and *J*_3_ represent height, width and length of video respectively.

**Figure 2:**
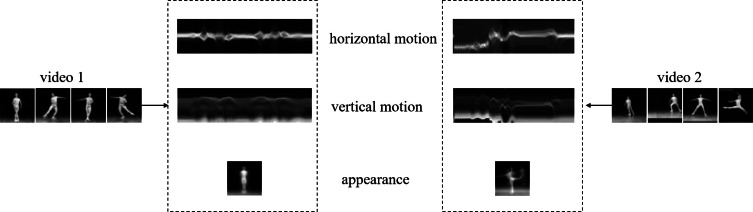
A visual example of matrix unfolding. Two videos with different labels are shown for comparison, which come from Ballet datasets (it will be discussed in ‘Experiments’). The two dashed frames show overlay horizontal motion, vertical motion and appearance of video 1 and video 2 respectively.

Before introducing data representation on PGM, we give a schematic of matrix unfolding for a third tensor in Fig. 1. The reader can refer to Kolda & Bader (2009) for more theory on tensor operation. For ease of understanding we give a corresponding example of two videos described by tensor in Fig. 2. We find that the corresponding unfolding matrix is discriminative for two videos with different labels, hence the multi-dimensional information of video tensor is worth mining for classification task.

In the following, we discuss the way to represent multi-dimensional data on PGM. The variation for each mode of a tensor 𝒜 ∈ ℝ^*J*_1_×⋯×*J*_*M*_^ can be captured by HOSVD (followed as Lui, 2012), which factorize tensor $\mathcal{A}$ using the orthogonal matrices in the following equation:

\begin{eqnarray*}\mathcal{A}=\mathcal{S}{\times }_{1}{\mathbf{V }}^{(1)}{\times }_{2}\cdots {\times }_{M}{\mathbf{V }}^{(M)} \end{eqnarray*}

where **V**^(*m*)^ ∈ ℝ^*J*_*m*_×*d*_*m*_^ (*m* =1 , …, *M*) are orthogonal matrices spanning the row space with the first *J*_*m*_ rows associated with non-zero singular values from the unfolded matrices respectively, 𝒮 ∈ ℝ^*d*_1_×⋯×*d*_*M*_^ is a core tensor, *d*_*m*_ = ∏_*i*≠*m*_*J*_*i*_, and ×_*m*_(*m* =1 ,…,*M*) denotes mode- *m* multiplication. Each **V**^(*m*)^^*T*^ ∈ ℝ^*d*_*m*_×*J*_*m*_^ is a tall orthogonal matrix. We take the first *p*_*m*_ (*p*_*m*_ ≤ *J*_*m*_) columns of **V**^(*m*)^^*T*^ and denote it as **U**^(*m*)^ ∈ ℝ^*d*_*m*_×*p*_*m*_^. Hence, **U**^(*m*)^ is a point on Grassmann manifold $\mathcal{G}({p}_{m},{d}_{m})$. And then (**U**^(1)^, …, **U**^(*M*)^) is a point on PGM $\mathcal{G}({p}_{1},{d}_{1})&times; &ctdot; &times; \mathcal{G}({p}_{M},{d}_{M})$.

Remark: The value of parameter *p*_*m*_(*m* =1 , …, *M*) reflects the principal information of data. In brief, the information of data may be redundant if the value of *p*_*m*_ is too large and the information of data may be insufficient if the value of *p*_*m*_ is too small. Hence it is important to select the parameters *p*_*m*_(*m* =1 , …, *M*) and we will discuss this problem in details in our experiments.

## Weighted sparse coding on product Grassmann manifold

### Weighted sparse coding model on PGM

Let {[**X**_1_], …, [**X**_*N*_]} be the training set which includes *N* samples, where $[{\mathbf{X}}_{i}]=({\mathbf{X}}_{i}^{1},\ldots ,{\mathbf{X}}_{i}^{M})\in \mathcal{PG}({p}_{1},\ldots ,{p}_{M}{|}{d}_{1},\ldots ,{d}_{M})$ is a point on product Grassmann manifold. Let $[\mathbf{Y }]=({\mathbf{Y }}^{1},&hellip; ,{\mathbf{Y }}^{M})&isin; \mathcal{PG}$(*p*_1_,…,*p*_*M*_|*d*_1_,…,*d*_*M*_) be a query sample on product Grassmann manifold. The sparse coding model on PGM is formulated as follows:

\begin{eqnarray*}\min _{\alpha }{d}_{\mathcal{PG}}^{2}([\mathbf{Y }],{\mathop{\biguplus }\nolimits }_{i=1}^{N}{\alpha }_{i}\odot [{\mathbf{X}}_{i}])+\lambda \parallel \alpha {\parallel }_{1} \end{eqnarray*}

where *α* = (*α*_1_, …, *α*_*N*_)^*T*^ is the sparse representation coefficient, the abstract symbols ${\mathop{\biguplus }\nolimits }_{i=1}^{N}$and ⊙ are used to simulate “linear” combination defined on PGM, *i.e.,* addition and scalar-mulitplication. ${d}_{\mathcal{PG}}([\mathbf{Y }],{\mathop{\biguplus }\nolimits }_{i=1}^{N}{\alpha }_{i}\odot [{\mathbf{X}}_{i}])$ measures the distance between reconstruction ${\mathop{\biguplus }\nolimits }_{i=i}^{N}{\alpha }_{i}\odot [{\mathbf{X}}_{i}]$ and the query sample [**Y**]. To get the sparse coding model on PGM, proper definitions of distance and combination operator should be specified. According to the geometric property of Grassmann manifold, we use the embedded distance and linear combination on the space of symmetric matrices. Hence, we construct the weighted sparse coding model on PGM as follows,

(1) \begin{eqnarray*}\min _{\alpha }\sum _{m=1}^{M}{\omega }_{m}\parallel {\mathbf{Y }}^{m}{{\mathbf{Y }}^{m}}^{T}-\sum _{i=1}^{N}{\alpha }_{i}({\mathbf{X}}_{i}^{m}{{\mathbf{X}}_{i}^{m}}^{T}){\mathop{\parallel }\nolimits }_{F}^{2}+\lambda \parallel \alpha {\parallel }_{1}.\end{eqnarray*}

### Algorithm for the weighted sparse coding on PGM

In this subsection, we show how to solve the optimization Eq. (1). We have

\begin{eqnarray*}\min _{\alpha }\sum _{m=1}^{M}{\omega }_{m}\parallel {\mathbf{Y }}^{m}{{\mathbf{Y }}^{m}}^{T}-\sum _{i=1}^{N}{\alpha }_{i}({\mathbf{X}}_{i}^{m}{{\mathbf{X}}_{i}^{m}}^{T}){\mathop{\parallel }\nolimits }_{F}^{2} &  =\min _{\alpha }\sum _{m=1}^{M}{\omega }_{m} \left\{ \right. \mathrm{Tr}({{\mathbf{Y }}^{m}}^{T}{\mathbf{Y }}^{m}{{\mathbf{Y }}^{m}}^{T}{\mathbf{Y }}^{m})+\sum _{i,j=1}^{N}{\alpha }_{i}{\alpha }_{j}\mathrm{Tr}({{\mathbf{X}}_{i}^{m}}^{T}{\mathbf{X}}_{j}^{m}{{\mathbf{X}}_{j}^{m}}^{T}{\mathbf{X}}_{i}^{m})\nonumber\\\displaystyle  -2\sum _{i=1}^{N}{\alpha }_{i}\mathrm{Tr}({{\mathbf{X}}_{i}^{m}}^{T}{\mathbf{Y }}^{m}{{\mathbf{Y }}^{m}}^{T}{\mathbf{X}}_{i}^{m}) \left( \right. . \end{eqnarray*}

For simplicity, we define a matrix *K*^*m*^(**X**) and a vector *K*^*m*^(**X**, **Y**) as following, *i.e.,* their elements are

$[{K}^{m}(\mathbf{X})]_{ij}={\omega }_{m}\mathrm{Tr}({{\mathbf{X}}_{i}^{m}}^{T}{\mathbf{X}}_{j}^{m}{{\mathbf{X}}_{j}^{m}}^{T}{\mathbf{X}}_{i}^{m}),~~i,$j=1 , …, *N*

$[{K}^{m}(\mathbf{X},\mathbf{Y })]_{i}={\omega }_{m}\mathrm{Tr}({{\mathbf{X}}_{i}^{m}}^{T}{\mathbf{Y }}^{m}{{\mathbf{Y }}^{m}}^{T}{\mathbf{X}}_{i}^{m}),~~i=1,\ldots ,N$

Hence the model Eq. (1) becomes

\begin{eqnarray*} & \min _{\alpha }\sum _{m=1}^{M} \left\{ {\alpha }^{T}{K}^{m}(\mathbf{X})\alpha -2{\alpha }^{T}{K}^{m}(\mathbf{X},\mathbf{Y }) \right\} +\lambda \parallel \alpha {\parallel }_{1} &  = & \min _{\alpha }{\alpha }^{T} \left( \sum _{m=1}^{M}{K}^{m}(\mathbf{X}) \right) \alpha -2{\alpha }^{T} \left( \sum _{m=1}^{M}{K}^{m}(\mathbf{X},\mathbf{Y }) \right) +\lambda \parallel \alpha {\parallel }_{1}. \end{eqnarray*}

The symemetric matrix $\sum _{m=1}^{M}{K}^{m}(\mathbf{X})$ is positive semidefinite since for all **v** = (*v*_1_, *v*_2_, …, *v*_*N*_)^*T*^ ∈ ℝ^*N*^:

\begin{eqnarray*}{\mathbf{v}}^{T} \left( \sum _{m=1}^{M}{K}^{m}(\mathbf{X}) \right) \mathbf{v} & =\sum _{m=1}^{M}\sum _{i=1}^{N}\sum _{j=1}^{N}{\omega }_{m}{v}_{i}{v}_{j}\mathrm{Tr}({{\mathbf{X}}_{i}^{m}}^{T}{\mathbf{X}}_{j}^{m}{{\mathbf{X}}_{j}^{m}}^{T}{\mathbf{X}}_{i}^{m})=\sum _{m=1}^{M}{\omega }_{m}\mathrm{Tr} \left( \sum _{i=1}^{N}\sum _{j=1}^{N}{v}_{i}{v}_{j}{{\mathbf{X}}_{i}^{m}}^{T}{\mathbf{X}}_{j}^{m}{{\mathbf{X}}_{j}^{m}}^{T}{\mathbf{X}}_{i}^{m} \right) =\sum _{m=1}^{M}{\omega }_{m}\parallel \sum _{i=1}^{N}{v}_{i}{\mathbf{X}}_{i}^{m}{{\mathbf{X}}_{i}^{m}}^{T}{\mathop{\parallel }\nolimits }_{F}^{2}\geq 0. \end{eqnarray*}

Therefore, the problem is convex and can be solved by a vectorized sparse coding problem. In detail, let **U**Σ**U**^*T*^ be the SVD of $\sum _{m=1}^{M}{K}^{m}(\mathbf{X})$, then the problem is equal to

(2) \begin{eqnarray*}\min _{\alpha }\parallel {\mathbf{Y }}^{\ast }-\mathbf{A}\alpha {\parallel }^{2}+\lambda \parallel \alpha {\parallel }_{1}\end{eqnarray*}

where **A** = Σ^1/2^**U**^*T*^ and ${\mathbf{Y }}^{\ast }={\Sigma }^{-1/2}{\mathbf{U}}^{T} \left( \sum _{m=1}^{M}{K}^{m}(\mathbf{X},\mathbf{Y }) \right) $. The pseudo-code for performing the proposed weighted sparse coding on PGM is summarized in Algorithm 1, which is simply called WSC-PGM.

 
_______________________ 
Algorithm 1 Weighted sparse coding on product Grassmann manifold (WSC- 
PGM)_______________________________________________________________________________________________ 
Require: 
   Training data includes N samples on PGM: [Xi] = (X1i,X2i,...,XMi), i = 
   1,2,...,N and Xmi ∈G(pm,dm), m = 1,2,...,M; the query sample on PGM: 
   [Y] = (Y1,Y2,...,YM) and Ym ∈G(pm,dm),m=1,2,...,M. 
Ensure: 
   The sparse code α∗ 
  for m = 1 : M do 
     for i = 1 : N do 
        for j = 1 : N do 
         [Km(X)]ij = ωmTr(XmiTXm 
j Xm 
j TXm 
i )       /* compute matrix Km(X) 
        end for 
         [Km(X,Y)]i  = ωmTr(XmiTYmYmTXm 
i )            /* compute vector 
   Km(X,Y) 
     end for 
  end for 
   M 
    ∑ 
 m=1 Km(X) = UΣUT          /* compute SVD of  M 
  ∑ 
m=1 Km(X) 
  A ← Σ1/2UT 
    Y∗ ← Σ−1/2UT ( M 
      ∑ 
  m=1 Km(X,Y)) 
  α∗ = arg min 
α  ∥Y∗− Aα∥2 + λ∥α∥1           /* the solution of model (2) 
  return  α∗_______________________________________________    

### Classification rule and algorithm

When model Eq. (1) is minimized, the optimal coefficient *α*^∗^ can be used for classification. Following the idea of the Sparse Representation Classification (SRC) (Wright et al., 2008), the query sample can be classified by it’s codes *α*^∗^ of these labeled training samples [**X**_*i*_]i =(1 , 2, …, *N*).

In details, let $({\alpha }_{1}^{\ast }\delta ({l}_{1}-k),{\alpha }_{2}^{\ast }\delta ({l}_{2}-k),\ldots ,{\alpha }_{N}^{\ast }\delta ({l}_{N}-k))^{T}$ be the class- *k* sparse codes, where *l*_*i*_(i =1 , 2, …, *N*) is the class label of training sample [**X**_*i*_] and *δ*(*x*) is the discrete Dirac function.

\begin{eqnarray*}\delta (x)= \left\{ \begin{array}{@{}cc@{}} \displaystyle 1&\displaystyle x=0\\ \displaystyle 0&\displaystyle else \end{array}. \right. \end{eqnarray*}

The residual error of a query sample [**Y**] =(**Y**^1^, **Y**^2^, …, **Y**^*M*^) by using the samples associated to class *k* is defined as

(3) \begin{eqnarray*}{}_{k}([\mathbf{Y }])=\sum _{m=1}^{M}{\omega }_{m}\parallel {\mathbf{Y }}^{m}{{\mathbf{Y }}^{m}}^{T}-\sum _{i=1}^{N}{\alpha }_{i}^{\ast }({\mathbf{X}}_{i}^{m}{{\mathbf{X}}_{i}^{m}}^{T})\delta ({l}_{i}-k){\mathop{\parallel }\nolimits }_{F}^{2}.\end{eqnarray*}

Then the estimated class of the query **Y** is determined by

(4) \begin{eqnarray*}\mathrm{Label}([\mathbf{Y }])=\arg \nolimits \min _{k}{}_{k}([\mathbf{Y }]).\end{eqnarray*}

The procedure of sparse representation classification on product Grassmann manifold is summarized in Algorithm 2.

 
________________________________________________________ 
Algorithm 2 Weighted sparse representation classification on product Grass- 
mann manifold (WSRC-PGM)_______________________________________________________________ 
Require: 
   Training data [Xi] = (X1i,X2i,...,XMi),i=1,2,...,N belonging to c classes; 
   the query [Y] = (Y1,Y2,...,YM) 
Ensure: 
   The class label Label([Y]) of the given test sample [Y] 
  Compute α∗ as Algorithm 1 
  Compute residual ɛk([Y]) by using equation (3) 
  Compute the class label by using equation (4) 
  return  Label([Y])___________________________________________________________________________    

## Experiments

In this section, we show performance of the proposed method against some state-of-the-art methods on three kinds of datasets. In the following experiments, all video data can be regarded as points on PGM $\mathcal{G}({p}_{1},{d}_{1})&times; \mathcal{G}({p}_{2},{d}_{2})&times; \mathcal{G}({p}_{3},{d}_{3})$ and the parameter *λ* is all chosen as 0.1 by experience.

### Cambridge hand gesture datasets

The Cambridge hand gesture datasets (Kim & Cipolla, 2008) contains 900 video sequences with 9 classes and it is divided into 5 sets according to different illuminations. The 9 classes are flat-leftward (FL), flat-rightward (FR), flat-contract (FC), spread-leftward (SL), spread-rightward (SR), spread-contract (SC), V-shape-leftward (VL), V-shape-rightward (VR) and V-shape-contract (VC) respectively. We follow the experimental protocol in paper (Kim & Cipolla, 2008), set 5 (normal illumination) is considered for training while the remaining sequences (with different illumination characteristics) are used for testing. In this experiment, the original sequences are converted to grayscale and resized to 20 × 20 × 20. Obviously, experiment results depend on the selection of parameters, so we firstly discuss the parameter setting in the following.

#### Parameter setting

In this subsection, we discuss the parameter setting including dimensions (*p*_1_, *p*_2_, *p*_3_) of three factor Grassmann manifolds and their weights (*ω*_1_, *ω*_2_, *ω*_3_). In fact, we have *ω*_1_ + *ω*_2_ + *ω*_3_ = 1 in model Eq. (1). Hence, we jointly determine the parameters (*p*_1_, *p*_2_, *p*_3_, *ω*_1_, *ω*_2_). For this datasets, *p*_1_, *p*_2_, *p*_3_ are optimized all in the range of 2 to 20 by step 2, and *ω*_1_, *ω*_2_ are optimized in the range as Table 2. We perform 5-fold cross validation on Set5 and find the optimal $({p}_{1}^{\ast },{p}_{2}^{\ast },{p}_{3}^{\ast },{\omega }_{1}^{\ast },{\omega }_{2}^{\ast })$ to obtain the best experimental results. Each time we leave one cross validation set as testing and the other four folds for training. Recursively, we perform experiments and record the correct recognition rate (CRR) of each fold.

**Table 2:** The range of *ω*_1_, *ω*_2_.

*ω*_1_∖*ω*_2_	0.1	0.2	0.3	0.4	0.5	0.6	0.7	0.8
0.1	(0.1, 0.1)	(0.1, 0.2)	(0.1, 0.3)	(0.1, 0.4)	(0.1, 0.5)	(0.1, 0.6)	(0.1, 0.7)	(0.1, 0.8)
0.2	(0.2, 0.1)	(0.2, 0.2)	(0.2, 0.3)	(0.2, 0.4)	(0.2, 0.5)	(0.2, 0.6)	(0.2, 0.7)	
0.3	(0.3, 0.1)	(0.3, 0.2)	(0.3, 0.3)	(0.3, 0.4)	(0.3, 0.5)	(0.3, 0.6)		
0.4	(0.4, 0.1)	(0.4, 0.2)	(0.4, 0.3)	(0.4, 0.4)	(0.4, 0.5)			
0.5	(0.5, 0.1)	(0.5, 0.2)	(0.5, 0.3)	(0.5, 0.4)				
0.6	(0.6, 0.1)	(0.6, 0.2)	(0.6, 0.3)					
0.7	(0.7, 0.1)	(0.7, 0.2)						
0.8	(0.8, 0.1)							

**Figure 3:**
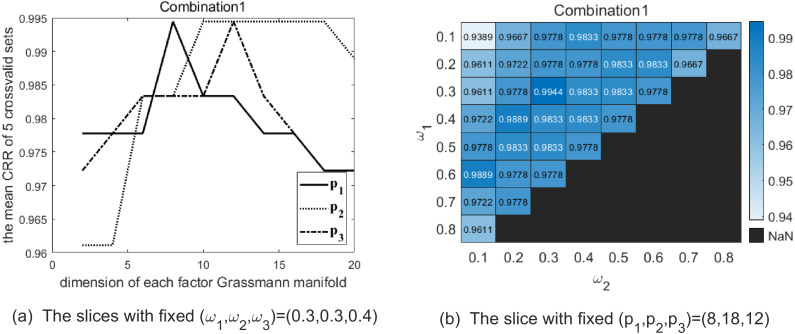
The two graphs show the slice of CRR’s variation with each parameter with combination 1 on the Cambridge Hand Gesture Datasets. (A) The solid line shows the variation of CRR with varying *p*_1_ while (*p*_2_, *p*_3_) are fixed as (18, 12), and the optimal *p*_1_ is 8 in this slice. The dotted line shows the variation of CRR with varying *p*_2_ while (*p*_1_, *p*_3_) are fixed as (8, 12), and the optimal *p*_2_ is 18 in this slice. The dash-dot line shows the variation of CRR with varying *p*_3_ while (*p*_1_, *p*_2_) are fixed as (8, 18), and the optimal *p*_3_ is 12 in this slice. (B) The heatmap reflects the variation of CRR with different (*ω*_1_, *ω*_2_) and the optimal (*ω*_1_, *ω*_2_) is (0.3, 0.3).

**Figure 4:**
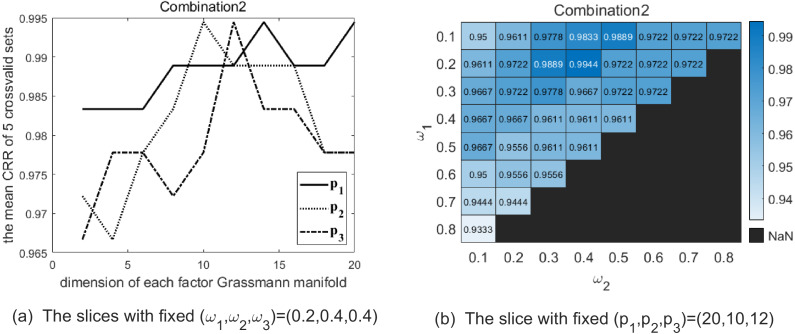
The two graphs show the slice of CRR’s variation with each parameter with combination 2 on the Cambridge Hand Gesture Datasets. (A) The solid line shows the variation of CRR with varying *p*_1_ while (*p*_2_, *p*_3_) are fixed as (10, 12), and the optimal *p*_1_ is 20 in this slice. The dotted line shows the variation of CRR with varying *p*_2_ while (*p*_1_, *p*_3_) are fixed as (20, 12), and the optimal *p*_2_ is 10 in this slice. The dash-dot line shows the variation of CRR with varying *p*_3_ while (*p*_1_, *p*_2_) are fixed as (20, 10), and the optimal *p*_3_ is 12 in this slice. (B) The heatmap reflects the variation of CRR with different (*ω*_1_, *ω*_2_), and the optimal (*ω*_1_, *ω*_2_) is (0.2, 0.4).

**Figure 5:**
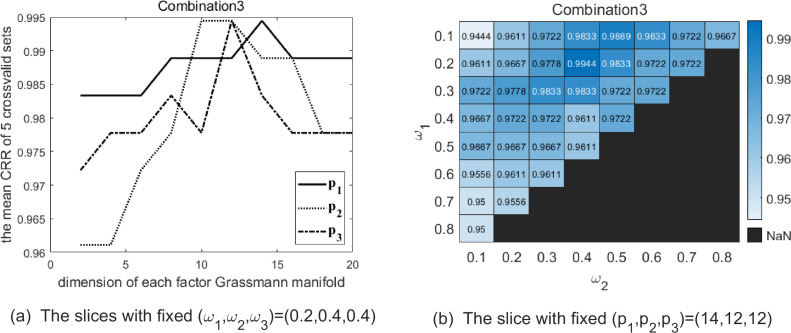
The two graphs show the slice of CRR’s variation with each parameter with combination 3 on the Cambridge Hand Gesture Datasets. (A) The solid line shows the variation of CRR with varying *p*_1_ while (*p*_2_, *p*_3_) are fixed as (12, 12), and the optimal *p*_1_ is 14 in this slice. The dotted line shows the variation of CRR with varying *p*_2_ while (*p*_1_, *p*_3_) are fixed as (14, 12), and the optimal *p*_2_ is 12 in this slice. The dash-dot line shows the variation of CRR with varying *p*_3_ while (*p*_1_, *p*_2_) are fixed as (14, 12), and the optimal *p*_3_ is 12 in this slice. (B) The heatmap reflects the variation of CRR with different (*ω*_1_, *ω*_2_) and the optimal (*ω*_1_, *ω*_2_) is (0.2, 0.4) in this slice.

Maximizing the average CRRs of five results to have good discrimination, there exist 33 optional parameter combinations. Meanwhile, we expect the data representation carrying more information to better fit the testing data. Hence, among the 33 combinations we choose the top 5 % combinations making *p*_1_ + *p*_2_ + *p*_3_ larger. We list the selected combinations of parameters (*p*_1_, *p*_2_, *p*_3_, *ω*_1_, *ω*_2_, *ω*_3_) in Table 3. Table 4 shows the CRRs of the five folds of Set5 with the combinations of parameter in Table 3. In order to illustrate the above parameter selection process, Figs. 3–5 show the slice of CRR’s variation with each dimension of parameter corresponding to the optimal combinations listed in Table 3, respectively.

#### Experiment result on testing sets

In this experiment, the parameter *λ* is set as 0.1. With the three combinations of parameters (*p*_1_, *p*_2_, *p*_3_, *ω*_1_, *ω*_2_), the samples of Set1-Set4 are represented as points on $\mathcal{PG}(8,18,12{|}400,400,400)$, $\mathcal{PG}(20,10,12{|}400,400,400)$ and $\mathcal{PG}(14,12,12{|}400,400,400)$ respectively. Table 5 summarizes the correct recognition rate for Set1-Set4 and the average correct recognition rate which followed by the standard deviation. As Table 5 shows, WSRC-PGM has superior performance compared with TCCA (Kim & Cipolla, 2008), PM (Lui, 2012), gSC and kgSC (Harandi et al., 2015), DMD+SC(SCCD2) (Singh et al., 2021).

**Figure 6:**
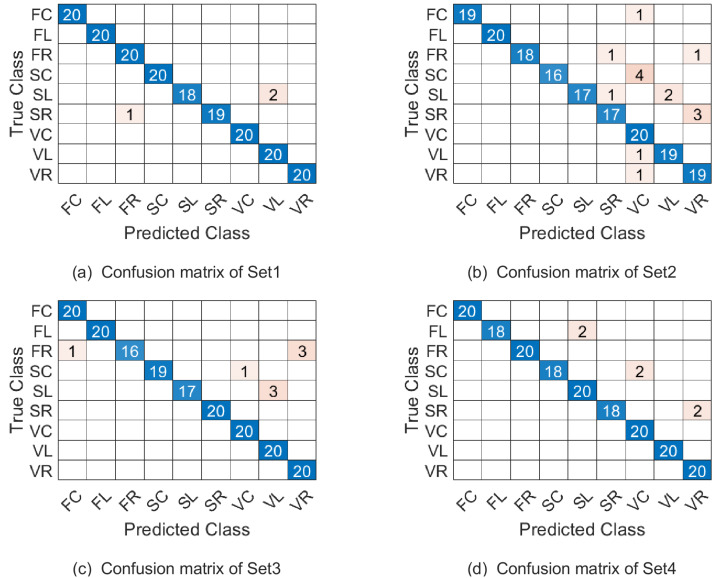
The confusion matrix of combination 1 on the Cambridge hand-gesture dataset.

The confusion matrix of our proposed approach on the four testing sets under parameter combination 1 are given in Fig. 6. Naturally, confusion matrices for combination 2 and 3 can be discussed similarly and they are omitted here. From Fig. 6 see, the most misclassified class is SL and most of the misclassified samples with lable SL were misassigned to the SC class. The second most misclassified class is SC and most of the misclassified samples with lable SC were misassigned to the VC class.

**Table 3:** The top 5% combinations of parameter on Cambridge hand gesture datasets.

Parameter	${p}_{1}^{\ast }$	${p}_{2}^{\ast }$	${p}_{3}^{\ast }$	${\omega }_{1}^{\ast }$	${\omega }_{2}^{\ast }$	${\omega }_{3}^{\ast }$
combination 1	8	18	12	0.3	0.3	0.4
combination 2	20	10	12	0.2	0.4	0.4
combination 3	14	12	12	0.2	0.4	0.4

**Table 4:** CRR of the five cross validation sets on Cambridge hand gesture datasets.

Cross validation sets	1	2	3	4	5
CRR of combination 1	100%	100%	100%	97.22%	100%
CRR of combination 2	100%	100%	100%	97.22%	100%
CRR of combination 3	100%	100%	100%	97.22%	100%

**Table 5:** Recognition results on the Cambridge hand-gesture dataset.

Method	Set1	Set2	Set3	Set4	Overall
TCCA (Kim & Cipolla, 2008)	81	81	78	86	82 ± 3.5%
PM (Lui, 2012)	93	89	91	94	91.7 ± 2.3%
gSC (Harandi et al., 2015)	93	92	93	94	93.3 ± 0.9%
kgSC (Harandi et al., 2015)	96	92	93	97	94.4 ± 2.0%
HOG3DVV+GGDA (Verma & Choudhary, 2018)	86	93	87	93	89.7
WSRC-PGM (combination 1)	98	92	96	97	95.6 ± 2.8%
WSRC-PGM (combination 2)	99	91	94	96	95.0 ± 3.5%
WSRC-PGM (combination 3)	99	89	94	96	94.3 ± 4.2%

### Ballet datasets

The Ballet dataset contains 44 videos including 8 complex motion patterns from 3 persons (Fathi & Mori, 2008). In detail, the actions are “left-to-right hand opening”, “right-to-left hand opening”, “standing hand opening”, “leg swinging”, “jumping”, “turning” , “hopping” and “standing still” . Main challenge of this dataset is large variations among classes such as speed, clothing and motion paths. The frame images are normalized and centered in a fixed size of 20 × 20. We extract total 2400 sub-videos by randomly sampling 6 frames from original video that exhibited the same action and then images are converted to grayscale. We randomly select 1200 samples as training set and the remainder as testing set.

**Figure 7:**
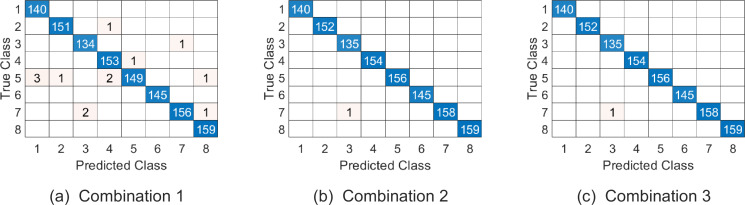
The confusion matrix of three combinations on Ballet datasets. The class labels “1-8” represent actions “left-to-right hand opening”, “right-to-left hand opening”, “standing hand opening”, “leg swinging”, “jumping”, “turning” , “hopping” and “standing still” respectively.

Similar to the discussion for parameter setting of experiment on Cambridge hand gesture dataset, we jointly determine the parameters (*p*_1_, *p*_2_, *p*_3_, *ω*_1_, *ω*_2_) by 5-fold cross validation on training set, where *p*_1_, *p*_2_ are all in the range of {2:2:20}, *p*_3_ is in the range of {1:1:6} and *ω*_1_, *ω*_2_ are in the range as Table 2. The top 5 % optional parameter combinations of (*p*_1_, *p*_2_, *p*_3_, *ω*_1_, *ω*_2_, *ω*_3_) are listed in Table 6. And the samples on testing set are represented on $\mathcal{PG}(10,6,2{|}120,120,400)$, $\mathcal{PG}(10,4,4{|}120,120,400)$ and $\mathcal{PG}(10,2,6{|}120,120,400)$ respectively in experiments. Table 7 summarizes the average correct recognition rate. The results show that our algorithm has superior performance compared with some state-of-the-art methods. And the confusion matrix of our proposed approach on the testing set under the three parameter combinations are given in Fig. 7.

**Table 6:** The top 5% combinations of parameter on Ballet dataset.

Parameter	${p}_{1}^{\ast }$	${p}_{2}^{\ast }$	${p}_{3}^{\ast }$	${\omega }_{1}^{\ast }$	${\omega }_{2}^{\ast }$	${\omega }_{3}^{\ast }$
combination 1	10	6	2	0.2	0.2	0.6
combination 2	10	4	4	0.2	0.2	0.6
combination 3	10	2	6	0.2	0.2	0.6

**Table 7:** Correct recognition rate on the Ballet dataset.

Method	CRR
(Fathi & Mori, 2008)	51%
DBoWs (Iosifidis, Tefas & Pitas, 2014)	91.1%
S-CTM (Wang & Mori, 2009)	91.36%
kgSC-dic (Harandi et al., 2015)	83.53 ± 0.8%
kgLC-dic (Harandi et al., 2015)	86.94 ± 1.1%
DMD+SC (SCCD2) (Singh et al., 2021)	96.25
WSRC-PGM (Combination 1)	98.9
WSRC-PGM (Combination 2)	99.9
WSRC-PGM (Combination 3)	99.9

### UMD Keck body-gesture datasets

The UMD Keck Body-Gesture Datasets contains 14 naval body gestures acquired from both static and dynamic backgrounds. The subjects and the camera remain stationary in the static backgrounds, the subjects and the camera are moving in the dynamic backgrounds. 126 videos and 168 videos are collected from the static scene and the dynamic environment respectively. The 14 body gestures are turn left, turn right, attention left, attention right, flap, stop left, stop right, stop both, attention both, start, go back, close distance, speed up and come near respectively.

We follow the experimental setting proposed in paper (Lin, Jiang & Davis, 2009). In the static background, we adopt Leave One Out Cross Validation (LOOCV). For dynamic background, the gestures acquired from the static background are used for training, while the gestures in dynamic background are used for testing.

**Figure 8:**
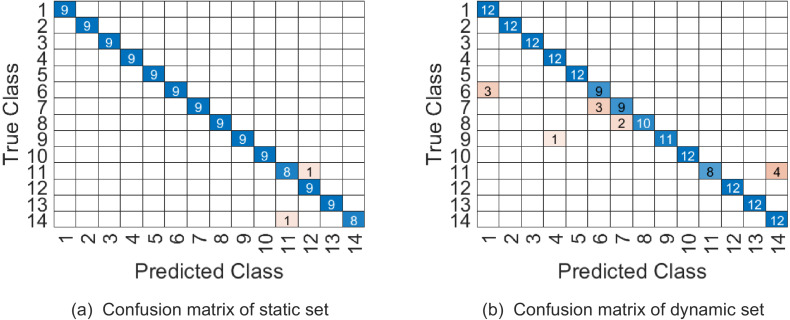
The confusion matrix of combination 1 on UMD Keck Body-Gesture datasets.

In our experiment, videos are firstly cropped by tracking the region of interest through a simple correlation filter, and then all videos are resized to 32 × 24 × 45. The videos whose frames are less than 45 are appended with the last frame added some Gaussian noise. Similar to the previous discussion, we jointly determine the parameters (*p*_1_, *p*_2_, *p*_3_, *ω*_1_, *ω*_2_) by 5-fold cross validation on training set, where *p*_1_ is in the range of {2:4:32}, *p*_2_ is in the range of {2:4:24}, *p*_3_ is in the range of {10:4:45} and (*ω*_1_, *ω*_2_) are in the range as Table 2. The top 5 % optional parameter combinations of (*p*_1_, *p*_2_, *p*_3_, *ω*_1_, *ω*_2_, *ω*_3_) are listed in Table 8. And the samples on testing set are represented on $\mathcal{PG}(6,22,14{|}1080,1440,768)$. Table 9 shows that WSRC-PGM has higher performance compared with TB (Lui, 2011), Prototype-Tree (Lin, Jiang & Davis, 2009) and PM (Lui, 2012). The confusion matrix of our proposed approach with parameter combination 1 are given in Fig. 8.

### Discussion

Through above experiments, we conclude that the proposed method is effective for video-based human gesture recognition. In experiments, the selection of parameters is a key step. We jointly selected optional parameters $({p}_{1}^{\ast },{p}_{2}^{\ast },{p}_{3}^{\ast },{\omega }_{1}^{\ast },{\omega }_{2}^{\ast },{\omega }_{3}^{\ast })$ on grid parameter set, through maximizing the average CRRs of 5-fold cross validation on training set. The parameter selection process is time-consuming because of the high dimension of parameter. This limitation may be solved by alternative iterations of optimization, through setting rational initial values based on prior information of data distribution. The reason is that the dimensions of parameter for each iteration can be reduced.

**Table 8:** The top 5% combinations of parameter on UMD Keck body-gesture dataset.

Parameter	${p}_{1}^{\ast }$	${p}_{2}^{\ast }$	${p}_{3}^{\ast }$	${\omega }_{1}^{\ast }$	${\omega }_{2}^{\ast }$	${\omega }_{3}^{\ast }$
combination 1	6	22	14	0.2	0.4	0.4

**Table 9:** Correct recognition rate on the UMD Keck Body-Gesture datasets.

Method	CRR of static	CRR of dynamic
TB (Lui, 2011)	92.1%	91.1%
Prototype-Tree (Lin, Jiang & Davis, 2009)	95.2%	91.1%
PM (Lui, 2012)	94.4%	92.3%
WSRC-PGM	98.4%	92.3%

## Computational complexity

We analyze the time complexity of WSC-PGM algorithm in this section. The algorithm focus on improving the correct recognition rate by sparse coding on product Grassmann manifold. Compared with sparse coding on single Grassmann manifold named as gSC (Harandi et al., 2015), we discuss the computation efficiency of WSC-PGM algorithm in the following.

Same as the notations of algorithm WSC-PGM, the WSC-PGM algorithm requires $O(N({d}_{1}{p}_{1}^{2}+{d}_{2}{p}_{2}^{2}+{d}_{3}{p}_{3}^{2}))$ flops for computing *K*^*m*^(**X**, **Y**). The gSC algorithm (Harandi et al., 2015) requires *O*(*Ndp*^2^) flops for computing ${\mathop{\parallel {\mathbf{Z}}^{T}{\mathbf{D}}_{j}\parallel }\nolimits }_{F}^{2}$j =1 , …, *N*, where span(**Z**), $\mathrm{span}({\mathbf{D}}_{j})&isin; \mathcal{G}(p,d)$ while other steps of the two algorithms have the same computational complexity. To make it easier for the readers to understand, we take the Cambridge Hand Gesture Dataset as an example, we set *d*_1_ = *d*_2_ = *d*_3_ = 400, *p*_1_ = 8, *p*_2_ = 18, *p*_3_ = 12 of combination 1 in our experiment and *d* = 400, *p* = 50 are chosen in gSC (Harandi et al., 2015). We can see that ${d}_{1}{p}_{1}^{2}+{d}_{2}{p}_{2}^{2}+{d}_{3}{p}_{3}^{2}=212800\ll d{p}^{2}=1000000$. However, the CRR of WSC-PGM algorithm is higher than that in gSC (Harandi et al., 2015).

We further evaluate the execution time of our WSC-PGM for classification in Table 10. And all experiments are executed on Intel(R) Core(TM) i7-10700 CPU with 32GB RAM.

**Table 10:** Time complexity(in seconds) for classifying testing sets on three datasets respectively.

	Cambridge hand gesture	Ballet	UMD Keck
PGM size	$\mathcal{PG}(8,18,12{|}400,400,400)$	$\mathcal{PG}(8,18,12{|}120,120,400)$	$\mathcal{PG}(6,22,14{|}1080,1440,768)$
Train size	180	1200	126
Test size	720	1200	168
Time	4.85	19.08	2.31

## Main Findings and Future Directions

Subject to video-based human gesture recognition, we proposed a novel weighted sparse coding model on product Grassmann manifold. A video can be viewed as a third order tensor and then represented as a point on product Grassmann manifold by factorizing the tensor through HOSVD. This representation can characterize the multi-dimensional information including appearance, horizontal motion, vertical motion from video data and also can efficiently take advantage of the nonlinear manifold structure of video data. Based on PGM representation of videos, we proposed a sparse coding method by embedding the product Grassmann manifold to the product space of symmetric matrices. Meanwhile, an efficient algorithm WSC-PGM and the corresponding classification algorithm WSRC-PGM are proposed. The method of this paper improves the correct recognition rate and meanwhile it reduces the time complexity comparing with sparse coding on single Grassmann manifold. Experiments on three kinds of public datasets show that our method performs very well.

In future work, we would like to study the product Grassmann manifold representation method combing with time series model in tensor form, in order to enhance the discriminant performance of videos.

## Additional Information and Declarations

### Data Availability

The following information was supplied regarding data availability: The code is available at GitHub: https://github.com/wangyp010/SRC_PGM. The data is available at: - Cambridge hand gesture database: https://labicvl.github.io/ges_db.htm
- Ballet dataset: https://www2.cs.sfu.ca/research/groups/VML/semilatent/
- UMD Keck body-gesture: http://www.zhuolin.umiacs.io/Keckgesturedataset.html.
